# A Multidimensional PERMA-H Positive Education Model, General Satisfaction of School Life, and Character Strengths Use in Hong Kong Senior Primary School Students: Confirmatory Factor Analysis and Path Analysis Using the APASO-II

**DOI:** 10.3389/fpsyg.2018.01090

**Published:** 2018-06-29

**Authors:** Man K. Lai, Cynthia Leung, Sylvia Y. C. Kwok, Anna N. N. Hui, Herman H. M. Lo, Janet T. Y. Leung, Cherry H. L. Tam

**Affiliations:** ^1^Applied Social Sciences, Hong Kong Polytechnic University, Kowloon, Hong Kong; ^2^Department of Applied Social Sciences, City University of Hong Kong, Kowloon, Hong Kong

**Keywords:** APASO-II, Hong Kong, affective and social outcomes, character strengths, confirmatory factor analysis, path analysis, PERMA-H, positive education

## Abstract

The multidimensional PERMA-H positive education model provided evaluation and education framework for the theoretical and practice development of positive psychology in schools. Character strengths use mediates the association of strength knowledge and well-being. Using the Assessment Program for Affective and Social Outcomes (2nd Version) (APASO-II), the Subjective Happiness Scale, and the Physical Health Subscale of the PERMA-profiler, a multidimensional measure of PERMA-H was validated using confirmatory factor analysis in the context of a positive education program evaluation in senior primary school students. The association of PERMA-H measurements with school well-being as measured by general satisfaction of school life, and levels of depression and anxiety, and the mediation mechanism of character strengths use in such association were studied using path analysis. A cross-sectional sample of 726 senior primary school students (i.e., grade 4–6) aged 8–13 from the two primary schools completed a baseline evaluation questionnaire of a positive education program. Satisfactory internal reliability of the scales was obtained with Cronbach's alpha coefficients < 0.70. The scales were generally positively and moderately inter-correlated, except for level of anxiety and depression symptoms which was negative. Good psychometric properties of APASO-II were evidenced from the factor structure of sub-scale scores conforming to six factors of the PERMA-H model by confirmatory factor analysis. Path analyses showed that the APASO-II factors together with measures of subject happiness and positive health as the multidimensional PERMA-H model of positive education differentially predicted general satisfaction of school life, level of anxiety and depression, and character strengths use. Character strengths use mediated the relationship of Positive Engagement with general satisfaction of school life. Positive education utilizes knowledge and research findings from positive psychology in schools to produce intended positive outcomes like enhanced well-being and reduced level of depression in students. This study provided a solid foundation for related scientific research and the understanding of the multidimensional framework of positive psychology concepts. Systematic promotion and longitudinal evaluation of positive education at the institutional level in Hong Kong can be achieved with the use of APASO-II and the positive education scales of subjective happiness and physical health.

## Introduction

Positive psychology is the “scientific study of optimal human functioning” (Linley et al., 2006, p. 8) and as a discipline, positive psychology studied positive emotion (i.e., happiness, joy, hedonia, subjective wellbeing/SWB, life with good things), engagement (i.e., flow, vitality, eudaimonia, psychological wellbeing/PWB, life with autonomy and actualization), and meaning (i.e., transcendence, purpose, life with connections) (Ryan and Deci, 2001; Seligman et al., 2009). This three components of Authentic Happiness theory was further developed into the five elements PERMA model (Seligman, 2011). Apart from the Positive Emotion (P), Engagement (E), and Meaning and Purpose (M), the two additional elements Relationships (R) and Achievement (A) covers wellbeing cultivate through human interactions and mastery goal pursuit (Norrish et al., 2013). These five unique elements of wellbeing are measurable, and wellbeing can be assessed under an integrative framework with a multidimensional understanding as proclaimed by the model. Wellbeing attained by optimal functioning is both holistic and multidimensional (Norrish et al., 2013). Individuals have the potentials to attain and experience wellbeing through various pathways and this connotes the multidimensional nature of wellbeing. The state of wellbeing experienced by oneself however would be a complete whole from all its related elements.

A multidimensional understanding of wellbeing provides theoretical and practical insights in the application of positive psychology at the different level of human organizations (Huppert and So, 2013; Kern et al., 2015). Although common core elements of wellbeing were evidenced across theories, countries and cultures, and individuals (Ryan and Deci, 2001; Peterson and Seligman, 2004; Park et al., 2006; Dodge et al., 2012; OCED, 2013), there are also variations in focus, levels, and pattern of relationships among elements of wellbeing (Ryff and Keyes, 1995) in which a profiler approach to assessed wellbeing and its application on the policies and programs (Huppert and So, 2013), interventions and trainings, and school individual students (Dodge et al., 2012; Kern et al., 2015; Butler and Kern, 2016) could be benefit.

Character strengths as vehicles to wellbeing are unique positive traits exist in every individuals which reflect in their thoughts, feelings, and behaviors to meet the challenges in the life and produce positive experiences (Peterson and Seligman, 2004). Derived from past cultural materials and recent literatures in wellbeing and happiness, twenty-four character strengths were identified and the Value in Action Inventory of Strengths (VIA-IS) was developed to measure these strengths. Good psychometric properties in large adult samples across countries (Park et al., 2006) and in different youth samples with the youth version Values in Action Inventory of Strengths for Youth (VIA-Youth) were found (Park and Peterson, 2006). The twenty-four character strengths are suggested to fall under six universal virtue categories, namely wisdom and knowledge, courage, humanity, justice, temperance, and transcendence (Peterson and Seligman, 2004). However, empirical studies did not consistently recover these six virtues (Peterson and Seligman, 2004; Shryack et al., 2010; Toner et al., 2012; Duan et al., 2013) and character strength measurements can be further developed and adapted into alternative measurement, such as the Chinese Virtues Questionnaire (CVQ) to reflect the other identified virtue structure and cultural emphases (Duan et al., 2013; Ho et al., 2014).

The investigation of universal virtues would be an important theoretical research question whereas the identification of a fix set of virtues might post challenges about individual potentials in producing positive experiences and acceptance in different groups of individuals. Apart from the approach of identifying latent virtues from manifested character strengths (Kristjánsson, 2012), another approach is to develop positive measurements from the PERMA model, as Butler and Kern's PERMA-Profiler (Butler and Kern, 2016) and Kern and colleagues' EPOCH Measure of Adolescent Well-Being (Kern et al., 2016). Pools of items were formed using items of scales used in wellbeing related literature and items of some character strengths sub-scales in the VIA-IS. The items were studied with calibration and validation subsets formed in the samples. Standard procedures in item selection, factor analyses of items to identify good performing items to the PERMA model structure, and development of good psychometric properties were performed in these PERMA-model-based positive measurements. This latter approach in developing positive measures for the study of wellbeing and evaluation of positive education programs has the advantage of a clear relationship between measurements and the elements of wellbeing, which can be complementary to the use of the twenty-four character strengths without a clear structure under the PERMA model (Oxford, 2016).

Apart from equipping strength knowledge, uses of character strengths also contribute to wellbeing and this is particularly important for positive psychology intervention to teach strengths use and evaluation studies to include this indicator in the understanding of the association between character strengths and wellbeing (Govinjdi and Linley, 2007; Quinlan et al., 2012), though character strengths predicted higher levels of wellbeing as indicated by vitality, positive affect, and perceived stress (Wood et al., 2011). Strengths use, like functioning, has a mediating nature in the process of strengths possessed to wellbeing outcomes (Weber et al., 2016). Evaluating its role through mediational analysis can provide information to the conceptual understanding of wellbeing development as well as education and evaluation practices in positive education.

The building of positive qualities in people and their lives apart from resolving negative issues in bio-psycho-social domains was herald among psychologists in the dawn of a new millennium (Seligman and Csikszentmihalyi, 2000). The absence of physical and mental illness is not sufficient for producing wellbeing, and assuming living can be without any negative emotions and experiences would be a fantasy. Signature character strengths in individuals can be identified, developed, and deployed (Seligman et al., 2009). Skills grounded in positive psychology theories and empirical findings can also be taught and developed to build individuals' positive qualities. This important breakthrough in the realization of implementing positive education in the schools, apart from what parents want and what schools teach, is a timely response to the general phenomenon of a high level of prosperity in the society but also a high prevalence of psychological issues, especially among the student population, in recent years (Seligman et al., 2009). In Hong Kong, the number of suicide cases in primary and secondary school doubled from around 10 cases each school year in 2013/14 and 2014/15, to around 20 cases each school year in 2015/16 and 2016/17 (Yip, 2016). A trend of increasing behavioral and emotional problems in students would catch the government's attention in trying to reduce such problems through positive education programs and a thorough rethinking of the educational goals and curriculum (Wu and Mok, 2017).

Positive Psychology Curriculum and Positive Psychology Intervention programs have been scientifically studied and implemented in a variety of school contexts, including the Penn Resiliency Program (PRP), Strath Haven Positive Psychology Curriculum (Seligman et al., 2009), Geelong Grammar School Applied Framework for Positive Education (Norrish, 2015a,b), and the St. Peter's College Positive Institution (White et al., 2015). Under the support of a local private foundation, a primary school in Hong Kong has launched a whole-school positive education program in 2016 utilizing the Model of Positive Education, which is based on Seligman's PERMA model plus a sixth element, the Positive Health, which embraces a holistic view of physical and psychological health through “practicing sustainable habits for optimal physical and psychological health” (Norrish et al., 2013, p. 155). Positive psychology does not only work at the student level, but also involving school administrators, teachers, and parents so that the school aim to become a positive education institution and community. Under the Model of Positive Education, positive education is learned through training to people in the ecology of students (Learn), taught as a curriculum (Teach), tied-in different school subjects, activities, and interactions (Embed), and applied in personal and work life (Live) so that students can acquire and master the way to flourish (Seligman et al., 2009; Norrish et al., 2013; Norrish, 2015a).

A program evaluation component was included in the program and positive measurements were used together with the government endorsed Assessment Program for Affective and Social Outcomes (APASO-II). The second version of the Assessment Program for Affective and Social Outcomes (APASO-II) was revised and launched in 2010/11 school year, for schools to examine their students' social and affective development and needs, and conduct self-evaluation on related program implemented in schools (Education Bureau HKSAR, 2016b). A wide range of items on numerous affective and social outcomes are selected to meet the aims and latest development of education in Hong Kong. Positive education has been receiving attention in the past few years and applications of positive psychology in kindergarten, primary and secondary schools, and also undergraduate teacher training have been reported in newspapers in recent years. The validated and developed APASO-II would be an invaluable tool in the development of positive education in Hong Kong.

The purpose of this study was to identify and confirm the appropriate use of Hong Kong government endorsed APASO-II as a positive psychology measurement under Seligman's PERMA (Seligman, 2011) and Norrish's PERMA-H models (Norrish et al., 2013; Norrish, 2015a) in a primary school implementing a whole-school positive education program. In parallel, the multidimensional PERMA-H positive education model was validated by a confirmatory factor analysis (CFA) with the identified APASO-II measurements, the Subjective Happiness Scale (SHS) for positive emotions (Lyubomirsky and Lepper, 1999), and Physical Health Subscale (PHS) of the PERMA-profiler for positive health (Butler and Kern, 2016) as the latter two dimensions were not captured by APASO-II subscales. Psychometric information of APASO-II subscales, SHS, PHS, and the Strength Use Questionnaire (Govinjdi and Linley, 2007; Wood et al., 2011), together with a measure on levels of anxiety and depression in students were presented. The PERMA-H dimensions would predict scores on general satisfaction of school life, levels of anxiety and depression, and strength use. Furthermore, it was hypothesized that strength use will have a mediating role in the association of positive measures with general satisfaction of school life and the mediation would be evaluated by path analysis in this study.

## Materials and methods

The main aim of the present study was to evaluate the association of selected APASO-II subscales guided by the PERMA model and Strengths Use Scale as having and using positive strengths with the General Satisfaction of school life subscale from APASO-II as an outcome in positive psychology among senior primary school students in a whole-school positive education program. The association with levels of anxiety and depression was also studied. Cross-sectional baseline survey data were collected with the scale items from the Chinese version of the scales or translated items with back-translated method. The dimensionality and internal consistency of these scales, and their correlations with anxiety and depression symptoms measured by the Hospital Anxiety and Depression Scale (Zigmond and Snaith, 1983; Leung et al., 1993), subjective happiness, and physical health were also presented as supporting evidence of the adequacy of using these scales for evaluation of positive education program.

APASO-II measurements for primary schools assess outcomes at four levels according to the bioecological model (Bronfenbrenner, 1995), namely self (as self-concept), self-others (as interpersonal relationships), self-school (as attitudes to school, motivation, causal attribution, learning competency, and independent learning capacity), and self-society (as values). The measurements are validated scales selected from the literature or developed by the government to represent various affective and social outcomes (Education Bureau HKSAR and The Hong Kong Institute of Education, 2010). For the evaluation research of an application of positive education in a local primary school, relevant subscales in the APASO-II were chosen by university professors with professional training in educational psychology and social work under the applied model for positive education (Norrish et al., 2013) developed from the multidimensional PERMA framework (Seligman, 2011; Kern et al., 2015) with an additional domain of Positive Health (Butler and Kern, 2016). Subscales in APASO-II representing character strengths of Positive Engagement include Perseverance, Success Effort Attribution, Effort Motivation, and Task Motivation; representing Positive Relationships include Parent Relationships, Peer Relationship, and Teacher-Student Relationship; representing Positive Purpose include Experience, Value of School Work, and Education Aims; representing Positive Accomplishment include Achievement and Academic Self Concept. Since no subscale of APASO-II indicates character strengths of Positive Emotions and Positive Health, the Subjective Happiness Scale (Lyubomirsky and Lepper, 1999) and the Physical Health Subscale of the PERMA-profiler (Butler and Kern, 2016) were adopted and an evaluation framework under the positive education model was formed to evaluate a Hong Kong whole-school positive education program.

Apart from the PERMA measurements adapted from the APASO-II, use of character strengths understood as another precursor of psychological well-being in primary school students was assessed by the Strengths Use Scale (Wood et al., 2011). Both having and using positive personal strengths lead to psychological well-being and the subscale General Satisfaction of School Life in APASO-II was the outcome measure of the positive education program.

### Sample

The studied sample in this study consists 726 primary 4 to 6 students with 436 (60.06%) students from the program school and 290 (39.94%) students from the comparison school. There were 232 (31.96%), 243 (33.47%), and 251 (34.57%) primary 4, 5, and 6 students, respectively. The percentages of boy and girl in the sample were 54.55% (396 students) and 45.45% (330 students). Students aged between 8 and 13 years old, with a mean and standard deviation of 9.93 and 0.91.

### Procedure

In the academic year of 2016/2017, a longitudinal three-year whole school positive education program has been implemented following the positive education model by Norrish (2015a). Positive education curriculums were developed by program personnel from the university and primary school according to the six pillars of character strengths through the four implementation levels of Learn It, Live It, Teach It, and Embed It. To study the effectiveness of the program, evaluation survey using positive psychology scales and APASO-II are administered in September of a year and June of the next year to capture any changes in the measurements. Another primary school of similar background to the program school but do not implement positive education program has formed the comparison group.

Students of the program school and comparison school were invited with parent and student consents obtained. The surveys are self-administered in classes without the presence of teachers but a research assistant to answer questions and assist students with problems in completing the survey. Students are instructed that participation is voluntary without any consequences and their identities are collected for matching the data at different timepoints. All information will be kept strictly confidential to the research personnel and only summary statistics will be used in reports and sharing of research findings.

Different versions of survey instruments are used for junior and senior primary school students, mainly due to their different literacy levels and age requirements of the scales. In the present study, we used only data from the senior primary school students of the program and comparison schools as the information collected from the survey were more comprehensive. A total of 791 questionnaires were distributed to the Primary 4 to 6 students of the two schools (486 and 305 from the program and comparison schools) and 788 questionnaires were collected. There were 570 students (72.34%) providing full information on the 115 items of the 17 studied measurements, age, and gender whereas the others were mostly missing only one item (115 students) from all measurements, or one item each in two to four measurements (36 students). In the calculation of scale scores, adopting a calculation allowing missing in less than 20% of the items (i.e., missing at most one item for every five items in a scale), the rate of completion would increase from 72.34% (570 students) to 92.13% (726 students). Analyses will be performed on responses of the 726 students providing information with less than 20% item missing in any single studied scale.

### Measures

#### Assessment programme for affective and social outcomes (2nd version) (APASO-II)

Developed in 2001 and revised in 2010, the Hong Kong government has adopted APASO as additional school quality assurance indicators on social and affective outcomes in students (Moore et al., 2006; Wu and Mok, 2017). Good validity and reliability evidences were established for APASO-II with a sample of 80,000 primary and 130,000 secondary students of 352 primary and secondary schools in Hong Kong, representing 36 and 29% of all primary and secondary school students (Education Bureau HKSAR, 2016a; Wu and Mok, 2017). Local norms were developed for schools to make reference in their self-evaluation and strategic planning. The APASO-II for primary schools consists eight scales which further separated into 53 subscales, and they are organized at four levels: self, self-others, self-school, and self-society (Education Bureau HKSAR and The Hong Kong Institute of Education, 2010).

For the present study in measuring strengths possessed by students, 12 subscales with 78 items were selected based on the four domains of Positive Engagement, Positive Relationships, Positive Purpose, and Positive Accomplishment in the positive education model. A thirteenth subscale, General Satisfaction under Quality of School Life was used as indicator for psychological well-being at school. Internal consistency coefficients (Cronbach's αs) of all the subscales were above 0.80 (Table 1). All items are rated on a Likert-type scale ranging from 1 (Not agree at all) to 4 (Extremely agree).

**Table 1:** Cronbach's αs of APASO-II subscales.

**Positive education domains**	**APASO-II subscales (please see Education Bureau HKSAR and The Hong Kong Institute of Education (2010) for simple descriptions of the subscales)**	**No. of items**	**αs**
Psychological well-being at school	General Satisfaction	6	0.94
Positive Engagement	Perseverance	10	0.91
	Success Effort Attribution	4	0.90
	Effort Motivation	7	0.92
	Task Motivation	4	0.87
Positive Relationships	Parent Relationships	8	0.92
	Peer Relationship	6	0.92
	Teacher-Student Relationship	7	0.95
Positive Purpose	Experience	5	0.83
	Value of School Work	5	0.90
	Education Aims	5	0.87
Positive Accomplishment	Achievement	6	0.93
	Academic Self Concept	5	0.88

#### Subjective happiness scale (SHS) and physical health subscale (PHS)

Apart from the APASO-II subscales, Positive Emotions and Positive Health of positive education model were assessed by the Subjective Happiness Scale (Lyubomirsky and Lepper, 1999) and the Physical Health Subscale of the PERMA-profiler (Butler and Kern, 2016). The Subjective Happiness Scale consists 4 items and are rated on a 7-point Likert-type scale. An item about “not very happy” was not used in this study as it is not about positive emotion. Internal consistency coefficient (Cronbach's α) of the three items was 0.87 in the current sample. The Physical Health Subscale consists 3 items about self-rated health using an 11-point Likert-type scale. Good internal consistency was observed in the current sample (Cronbach's α = 0.92).

#### Strengths use scale (SUS)

Use of strengths has been proposed as another important component in the study of positive psychology and well-being, and the Strengths Use Scale (Govinjdi and Linley, 2007; Wood et al., 2011) was validated and tested showing good reliability (Cronbach's α > 0.90) and construct validity. This scale consists 14 items to be rated on a 7-point Likert-type scale ranging from 1 (Strongly disagree) to 7 (Strongly agree). In the studied sample, good internal consistency (Cronbach's α = 0.97) was achieved.

#### Hospital anxiety and depression scale (HADS)

The Hospital Anxiety and Depression Scale was developed and used to assess states of anxiety and depression in medical settings (Zigmond and Snaith, 1983) and a Chinese version was developed showing good agreement with the original version as well as good psychometric properties (Leung et al., 1993). In this study, HADS would indicate ill-being and an expected negative correlation with other positive measures would provide discriminative validity evidence for the positive measures (Kern et al., 2015). Good internal reliability (Cronbach's alpha = 0.81) and concurrent validity with suicidal thought intensity in a large Hong Kong adolescent sample was reported (Chan et al., 2010). A Cronbach's α of 0.80 was found in the studied sample.

### Data analysis

Good psychometric properties of APASO-II were evaluated by internal consistency of subscales and the structure of domains under positive education model which was evaluated by confirmatory factor analysis (CFA). Cronbach's α value larger than 0.70 indicates good reliability of items of a measurement (Nunnally, 1978) and all subscales achieved 0.80 or above in Cronbach's α. Usefulness of APASO-II in positive education programs was evaluated by correlation analysis of positive education domain scores as mean scores of positive education domains with mean scores of SUS, HADS, and the General Satisfaction subscale of APASO-II. Positive moderate correlations between domains of positive education model, strengths use, and general satisfaction of school life, but negative correlations with symptoms and severity of anxiety and depression were expected. The factor structure of the PERMA-H positive education model was assessed by CFA under Structural Equation Modeling (SEM). Individual item scores of SHS and PHS for Positive Emotions (P) and Positive Health (H), and mean APASO-II sub-domain scores for Positive Engagement (E), Positive Relationships (R), Positive Purpose (M), and Positive Accomplishment (A) were loaded on their respective latent factors with factor variance fixed at 1, hence factor loadings could be freely estimated. Due to the nonnormality of manifested variables in the CFA model, robust Maximum-Likelihood estimation were performed using the asymptotic covariance matrix in LISREL (Jöreskog and Sörbom, 2001) and the Satorra-Bentler scaled χ^2^ would be calculated (Satorra and Bentler, 1994; Jöreskog and Sörbom, 2001).

A path model was hypothesized to describe the association between the six positive education domains with strengths use, general satisfaction, and anxiety and depression intensity. The multidimensional understanding of well-being as measured by general satisfaction of school life could be studied from the specific associations with the different positive education domains. Scores of Positive Emotions and Positive Health were represented by mean scores of SHS and PHS. Scores of the other four domains of positive education model, namely Positive Engagement, Positive Relationships, Positive Purpose, and Positive Accomplishment were calculated by the mean scores of the APASO-II subscales under the respective domains. The role of strengths use proposed by Wood et al. (2011) was tested as a mediator in the association of positive education strengths and school well-being in the path model as well.

The CFA of APASO-II subscales, SHS, and PHS under PERMA-H domains of positive education, and the path model of positive education mechanism were performed using LISREL (Jöreskog and Sörbom, 2001), whereas the other analyses were performed using SPSS 22.0 (IBM Corp, 2013). The χ^2^ test in Structural Equation Modeling is sensitive to sample size and is not appropriate as an absolute standard for evaluating models with large sample size (Bentler and Bonett, 1980). Models with 400 or more cases would almost always obtained a statistically significant χ^2^ (Kenny, 2015). In this current CFA and path analysis in a sample of over 700 students, other fit information was suggested in the evaluation of model fits (Wheaton et al., 1977; Hu and Bentler, 1998; Schermelleh-Engel et al., 2003). Goodness-of-fit indices and criteria for good fit in models would be indicated by the χ^2^/df (between 2 and 3), Comparative Fit Index (CFI close to or above 0.95), Tucker-Lewis coefficient (TLI close to or above 0.95), Standard Root Mean Square Residual (SRMR close to or below 0.08), and Root Mean Square Error of Approximation (RMSEA close to or below 0.06).

## Results

Among the thirteen APASO-II subscales, including the outcome subscale of General Satisfaction, SHS, PHS, HADS, and SUS, significant higher mean scores in girls were found in General Satisfaction, Task Motivation, Peer Relationships, Teacher-student relationship, Experience, Values of School Work, and Education Aims, but a significant lower mean score in HADS. There was no significant correlation between age and the studied variables.

Correlation coefficients among the studied APASO-II subscales, SHS, PHS, and SUS were generally positive and with strength of association from moderate (minimum *r* = 0.29) to large (*r* = 0.74). The correlation coefficients of HADS with the studied APASO-II subscales, SHS, PHS, and SUS were all negative, ranged from −0.49 to −0.31. This is consistent with the understanding that strength possessed and strengths use measurements under the positive education model are positively correlated whereas they are negatively correlated with states of anxiety and depression among the senior primary school students.

**Figure 1:**
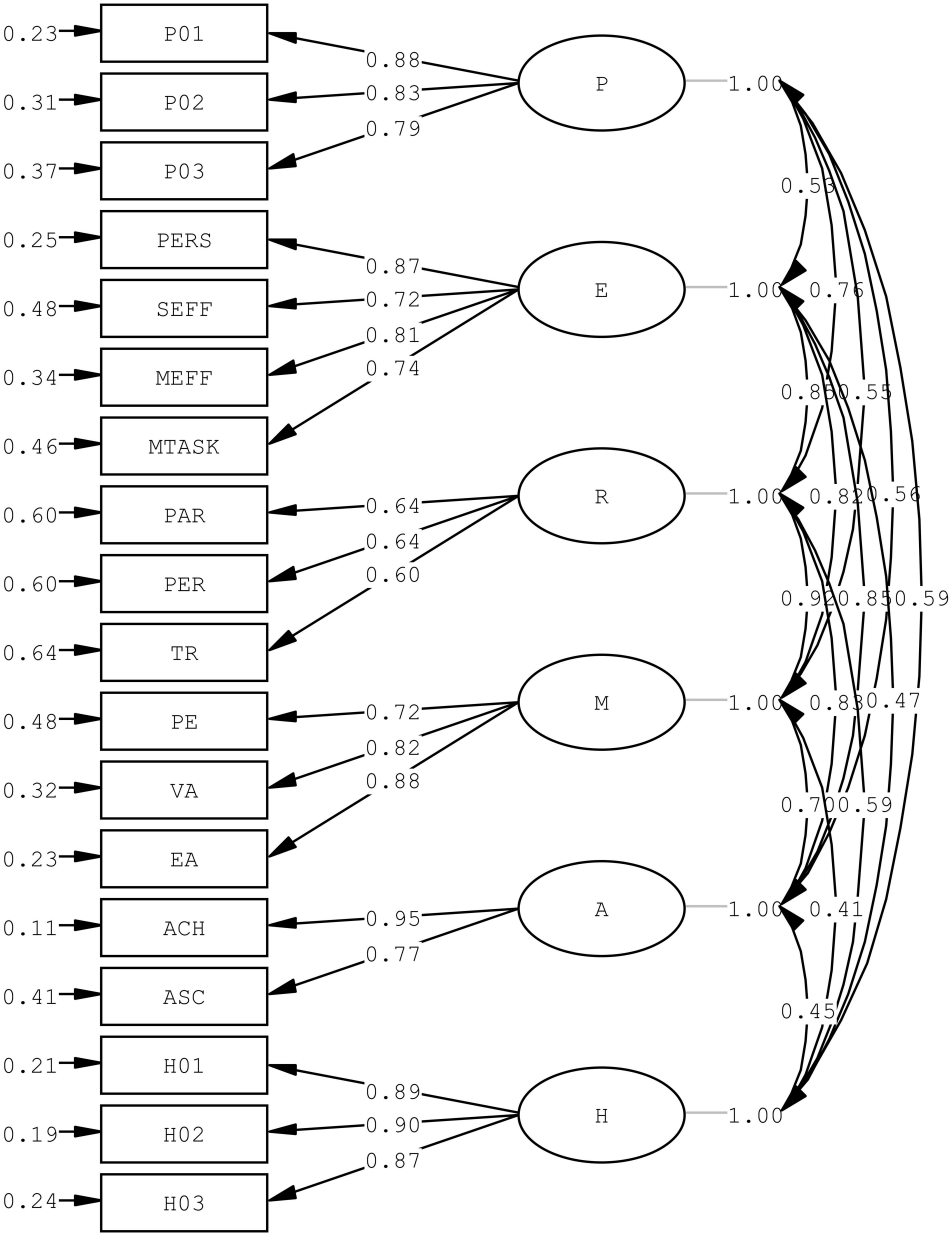
Confirmatory factor analysis of PERMA-H Positive Education Model with Positive Education Domains: (i) Positive Emotions manifested by the three Subject Happiness Scale (SHS) items, (ii) Positive Engagement (E), Positive Relationships (R), Positive Meaning (M), and Positive Achievement (A) manifested by the twelve the Assessment Program for Affective and Social Outcomes (2nd Version) (APASO-II) subscales, namely Perseverance (PERS), Success Effort Attribution (SEFF), Effort Motivation (MEFF), Task Motivation (MTASK), Parent Relationships (PAR), Peer Relationship (PER), Teacher-Student Relationship (TR), Experience (PE), Value of School Work (VA), Education Aims (EA), Achievement (ACH), and Academic Self Concept (ASC), and (iii) Positive Health by the three Physical Health Subscale (PHS) items.

Apart from the good support of scale internal reliability (Cronbach's αs >0.80) for the studied variables, CFA was performed on the PERMA-H positive education model with twelve APASO-II subscales (excluding the outcome variable General Satisfaction), SHS, and PHS to confirm the structure of positive education model of these APASO-II subscales. The positive education domains of Positive Engagement, Positive Relationships, Positive Purpose, and Positive Accomplishment manifested by the twelve APASO-II subscales and schematized in Table 1, and the Positive Emotions and Positive Health measurement items produced adequate goodness-of-fit, although a significant χ^2^ was found [Satorra-Bentler scaled χ${}_{\left(120\right)}^{2}$ = 306.50, *p* < 0.05]. The positive education model of six positive education domains generated a χ^2^/df of 2.55, CFI of 0.96, a TLI of 0.95, an SRMR of 0.04, and an RMSEA of 0.06. Factor loadings from APASO-II subscales to positive education domains were all positive and significant, ranging from 0.60 to 0.95 (Figure 1). The domains also correlated moderately to strongly among themselves (*r*s from 0.41 to 0.92).

**Figure 2:**
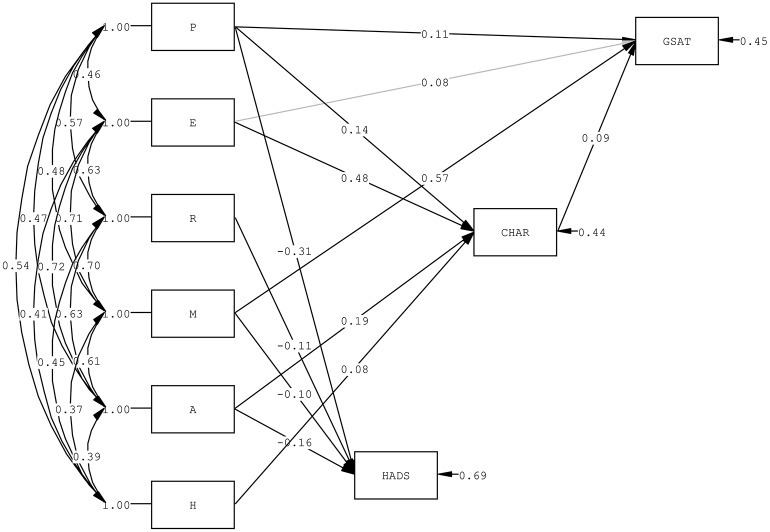
Path model of possessed strength under the multidimensional positive education model (PERMA-H) and outcomes of General Satisfaction (GSAT) and Hospital Anxiety and Depression Scale (HADS), with strengths use (CHAR) as a mediator.

Together with the Positive Emotions and Positive Health, the PERMA-H positive education model as strength possessed and the mediating role of strengths use in relationship with the outcome variable of General Satisfaction under Quality of School Life, and the direct association between PERMA-H domains with state of anxiety and depression were evaluated with path analysis. Firstly, outcomes of General Satisfaction and HADS, and the mediator strengths use were regressed on the six domains of the positive education model. This path model produced a good fit with the sample covariance matrix with a Satorra-Bentler scaled χ${}_{\left(3\right)}^{2}$ = 6.86 (*p* = 0.08), a χ^2^/df of 2.29, a CFI of 1.00, a TLI of 0.98, an SRMR of 0.01, and an RMSEA of 0.04. The positive education domains of Positive Emotions, Positive Engagement, and Positive Meaning significantly correlated with General Satisfaction. Positive Emotions, Positive Relationships, Positive Meaning, and Positive Achievement correlated negatively with HADS. Positive Emotions, Positive Engagement, Positive Achievement, and Positive Health correlated positively with strengths use. All positive education domains correlated positively among themselves (*r*s ranging from 0.37 to 0.72), supporting a general flourishing concept described with the different positive strengths. Secondly, the path model was estimated with the non-significant paths from positive education domains to outcomes and strengths use removed, resulting in a good model fit of Satorra-Bentler scaled χ${}_{\left(10\right)}^{2}$ = 12.25 (*p* = 0.27), χ^2^/df = 1.23, CFI = 1.00, TFI = 0.99, SRMR = 0.01, and RMSEA = 0.02. Lastly, the mediating role of strengths use in the association of positive education domains, namely Positive Emotions and Positive Engagement with General Satisfaction were evaluated by path analysis and a marginally significant change in χ^2^ was found [Δ in Satorra-Bentler scaled χ^2^_(1)_ = 3.84, *p* = 0.05] when General Satisfaction was also regressed on strengths use (Figure 2). This mediation model produced a good fit [χ${}_{\left(9\right)}^{2}$ = 9.15 and *p* = 0.42, χ^2^/df = 1.11, CFI = 1.00, TFI = 1.00, SRMR = 0.01, and RMSEA = 0.005]. Strengths use fully mediated the relationship of Positive Engagement with General Satisfaction. The positive education model of PERMA-H manifested by APASO-II subscales and happiness and physical health scales appropriately represented flourishing in senior primary school students. This multidimensional understanding of well-being correlated significantly and positively with general satisfaction of school life and strengths use, and negatively with state of anxiety and depression. Furthermore, the mediating role of strengths use was identified by the path analyses in the relationship of Positive Engagement with general satisfaction of school life.

## Discussion

Assessment Programme for Affective and Social Outcomes (2nd version) (APASO-II), a Hong Kong government endorsed outcome measures in education, was found to be an appropriate positive education measurement. Subscales of APASO-II conforms to four elements of the PERMA-H model, which were Positive Engagement, Positive Relationships, Positive Purpose, and Positive Accomplishment, as evidenced by a good model fit in the confirmatory factor analysis. One of its subscale, General Satisfaction is also an appropriate positive measure for wellbeing. All the thirteen selected subscales of APASO-II and the Strengths Use Scale (SUS) showed satisfactory internal consistency reliability of over 0.80. These positive education measures, together with Subjective Happiness Scale (SHS) and Physical Health Subscale (PHS) also correlated positively moderately with each other, and negatively with levels of anxiety and depression measured by Hospital Anxiety and Depression Scale (HADS). The APASO-II subscales together with the SHS and PHS validated a complete multidimensional PERMA-H positive education model (Seligman et al., 2009; Norrish, 2015a) and formed a positive education measurement in a primary school implementing a whole-school positive education program. Positive measures can be reliably assessed in Hong Kong primary school students as small as Primary 4 of eight years old. The multidimensional understanding would allow students to utilize signature strengths and address the other strengths, improve and develop strengths through Positive Psychology Intervention activities at schools (Kern et al., 2015). White et al. (2015) stated eight operational goals for an institution to turn into a positive institution and three of them are 1) a definition and measurement of wellbeing, 2) scientifically informed implementation of positive education program, and 3) evaluation of efficiency of the program. The PERMA-H model informed positive measures studied in this paper would help to prepare the positive education school to become a positive institution.

The mediation model of the relationships between positive measures informed by the PERMA-H model and wellbeing measures of general satisfaction of school life and levels of anxiety and depression mediated by strengths use was confirmed by the path analysis. Positive measures under the elements of Positive Emotion, Positive Engagement, Positive Achievement, and Positive Health predicted strengths use, whereas Positive Emotion, Positive Engagement, and Positive Purpose predicted general satisfaction of school life. Full mediation on the association between Positive Engagement and general satisfaction by strengths use was found. Except for Positive Relationships, the six dimensions of wellbeing measures directly or indirectly predicted general satisfaction of school life in the studied group of primary school students. Positive Relationships, together with Positive Emotion and Positive Achievement, showed negative associations with levels of anxiety and depression among the students. The dimension of relationship was emphasized in the Chinese culture (Ho et al., 2014) and cultural sensitive strength measures also identified this important dimensions (Duan et al., 2013). It would be interesting explore further if Positive Relationship would have a differential association with wellbeing at different development stages (Martínez-Martí and Ruch, 2014).

After more than a decade development of positive psychology, advancements in the models and theories, measurements and intervention programs, and actual whole-school implementation have been accumulated. School implementation of positive education in Hong Kong has been also getting popular. The finding of APASO-II subscales conforming to a positive education model would create possibilities for all schools in Hong Kong to evaluate their school programs under a positive education model and a strength-based approach. Academic achievement together with positive education outcomes can be assessed regularly in the schools and longitudinal assessment of these outcomes in education can inform education policies, programs, and management as well as the activities, the curriculum, and teaching and learning (Gilman and Huebner, 2003). This potential however is still not optimally actualized as schools are free to decide how frequent, which subscales used, and the students in the APASO-II administration.

The positive education model extends the application of positive psychology from a thriving individual to a thriving school community (Seligman et al., 2009; Norrish et al., 2013; Norrish, 2015b; White et al., 2015; Butler and Kern, 2016; MacIntyre, 2016). The strengths language can be shared among stakeholders of the school community and there will be a connection between students and the school community. The purpose of flourishing and utilization of character strengths can meet the opportunities for practice positive knowledge and skills provided in the school community (Noble and McGrath, 2015). The wellbeing of students would be hinged on positive interaction with school stakeholders and the community they are growing up and learning. The school as a positive institution would be the most appropriate environment for acquiring positive knowledge and skills, and to practice and live with these positive characters. APASO-II is a valid and reliable positive education measure and can be used with scales on Positive Emotion and Positive Health to represent the positive education model. This multidimensional measure creates possibilities for the longitudinal assessment of wellbeing in students, evaluation of school programs, and informing school policies and the development of a positive school community. Through regular and complete collection of APASO-II information, institution level longitudinal positive education information can be accumulated for theoretical and practical scientific research in positive education and positive psychology.

This study has several limitations. The psychometric properties and the mediation model were based on cross-sectional data with missing values, although the problem of missing values was circumvented by calculating mean scores with missing in less than 20% of the items in individual scales. The findings were also based on students studying senior primary school forms. APASO-II for secondary school is also available and the government encourages secondary schools to use it for the study of school performance. The multidimensional understanding of APASO-II subscales under the PERMA-H positive education model should be evaluated empirically. Validation study of APASO-II and other existing positive education and positive psychology measurements should be conducted to obtain a more comprehensive understanding of student wellbeing, character strengths and strengths use, the structure of virtue and PERMA-H domains, and the relationship among them. Existing Chinese version of such measures, specifically the Chinese Virtues Questionnaire (Duan et al., 2013) and the Flourishing Scale (Diener et al., 2010; Duan and Xie, 2016), can further anchor cultural specific domains from the core domains in wellbeing.

## Ethics statement

This study was carried out in accordance with the recommendations of guidelines of the Human Subjects Ethics Sub-committee (HSESC) with written informed consent from all subjects. All subjects gave written informed consent in accordance with the Declaration of Helsinki. The protocol was approved by the Human Subjects Ethics Sub-committee (HSESC).

## Author contributions

ML, CL, and SK contributed to the instrument selection and development, conceptualization, and drafting of the paper. ML contributed to the data analyses and interpretation of results. SK, CL, AH, HL, JL, and CT contribute to the conception and design of the study, acquisition of the data.

### Conflict of interest statement

The authors declare that the research was conducted in the absence of any commercial or financial relationships that could be construed as a potential conflict of interest.

## References

[B1] Bentler P. M. Bonett D. G. (1980). Significance tests and goodness of fit in the analysis of covariance structures. Psychol. Bull. 88, 588–606. 10.1037/0033-2909.88.3.588

[B2] Bronfenbrenner U. (1995). The bioecological model from a life course perspective: reflections of a participant observer, in Examining Lives in Context: Perspectives on the Ecology of Human Development, eds Moen P. Glenn J. Elder H. Luscher K. (Washington, DC: American Psychological Association), 619–647.

[B3] Butler J. Kern M. L. (2016). The PERMA-profiler: a brief multidimensional measure of flourishing. Int. J. Wellbeing 6, 1–48. 10.5502/ijw.v6i3.526

[B4] Chan Y.-F. Leung D. Y. P. Fong D. Y. T. Leung C.-M. Lee A. M. (2010). Psychometric evaluation of the hospital anxiety and depression scale in a large community sample of adolescents in Hong Kong. Qual. Life Res. 19, 865–873. 10.1007/s11136-010-9645-1 20373037 PMC2892613

[B5] Diener E. Wirtz D. Tov W. Chu K.-P. Choi D.-W. Oishi S. (2010). New well-being measures: short scales to assess flourishing and positive and negative feelings. Soc. Indic. Res. 97, 143–156. 10.1007/s11205-009-9493-y

[B6] Dodge R. Daly A. P. Huyton J. Sanders L. D. (2012). The challenge of defining wellbeing. Int. J. Wellbeing 2, 222–235. 10.5502/ijw.v2i3.4

[B7] Duan W. Ho S. M. Y. Bai Y. Tang X. (2013). Psychometric evaluation of the Chinese virtues questionnaire. Res. Soc. Work Pract. 23, 336–345. 10.1177/1049731513477214

[B8] Duan W. Xie D. (2016). Measuring adolescent flourishing: psychometric properties of flourishing scale in a sample of Chinese adolescents. J. Psychoeduc. Assess. 10.1177/0734282916655504. [Epub ahead of print].

[B9] Education Bureau HKSAR The Hong Kong Institute of Education (2010). Workshop on “Application of the Assessment Program for Affective and Social Outcomes (2nd Version)” Training Manual (For Primary Schools). Available online at: http://www.edb.gov.hk/en/sch-admin/sch-quality-assurance/performance-indicators/apaso2/training.html.

[B10] Education Bureau HKSAR (2016a). APASO-ll- Frequently Asked Questions. Available online at: http://www.edb.gov.hk/en/sch-admin/sch-quality-assurance/performance-indicators/apaso2/faq.html.

[B11] Education Bureau HKSAR (2016b). Assessment Program for Affective and Social Outcomes (2nd Version) (APASO-II). Available online at: http://www.edb.gov.hk/en/sch-admin/sch-quality-assurance/performance-indicators/apaso2/index.html.

[B12] Gilman R. Huebner S. (2003). A review of life satisfaction research with children and adolescents. Sch. Psychol. Q. 18, 192–205. 10.1521/scpq.18.2.192.21858

[B13] Govinjdi R. Linley P. A. (2007). Strengths use, self-concordance and well-being: implications for strengths coaching and coaching psychologists. Int. Coach. Psychol. Rev. 2, 141–153.

[B14] Ho S. M. Y. Duan W. Tang S. C. M. (2014). The psychology of virtue and happiness in Western and Asian thought, in The Philosophy and Psychology of Character and Happiness, eds Snow N. E. Trivigno F. V. (New York, NY: Routledge), 215–238.

[B15] Hu L.-T. Bentler P. M. (1998). Fit indices in covariance structure modeling: sensitivity to underparameterized model misspecification. Psychol. Methods 3, 424–453. 10.1037/1082-989X.3.4.424

[B16] Huppert F. A. So T. T. C. (2013). Flourishing across Europe: application of a new conceptual framework for defining well-being. Soc. Indic. Res. 110, 837–861. 10.1007/s11205-011-9966-7 23329863 PMC3545194

[B17] IBM Corp (2013). IBM SPSS Statistics for Windows (Version 22.0). Armonk, NY: IBM Corp.

[B18] Jöreskog K. G. Sörbom D. (2001). LISREL 8.5 for Windows. Lincolnwood, IL: Scientific Software International, Inc.

[B19] Kenny D. A. (2015). Measuring Model Fit. Available online at: http://davidakenny.net/cm/fit.htm.

[B20] Kern M. L. Benson L. Steinberg E. A. Steinberg L. (2016). The EPOCH measure of adolescent well-being. Psychol. Assess. 28, 586–597. 10.1037/pas0000201 26302102

[B21] Kern M. L. Waters L. E. Adler A. White M. A. (2015). A multidimensional approach to measuring well-being in students: application of the PERMA framework. J. Posit. Psychol. 10, 262–271. 10.1080/17439760.2014.936962 25745508 PMC4337659

[B22] Kristjánsson K. (2012). Positive psychology and positive education: old wine in new bottles? Educ. Psychol. 47, 86–105. 10.1080/00461520.2011.610678

[B23] Leung C. M. Ho S. Kan C. S. Hung C. H. Chen C. N. (1993). Evaluation of the Chinese version of the Hospital anxiety and depression scale. A cross-cultural perspective. Int. J. Psychosom 40, 29–34. 8070982

[B24] Linley P. A. Joseph S. Harrington S. Wood A. M. (2006). Positive psychology: past, present, and (possible) future. J. Posit. Psychol. 1, 3–16. 10.1080/17439760500372796

[B25] Lyubomirsky S. Lepper H. S. (1999). A measure of subjective happiness: preliminary reliability and construct validation. Soc. Indic. Res. 46, 137–155. 10.1023/A:1006824100041

[B26] MacIntyre P. D. (2016). So far so good: An overview of positive psychology and its contributions to SLA, in Positive Psychology Perspectives on Foreign Language Learning and Teaching, eds Gabryś-Barker D. Gałajda D. (Basel: Springer International Publishing), 3–20.

[B27] Martínez-Martí M. L. Ruch W. (2014). Character strengths and well-being across the life span: data from a representative sample of German-speaking adults living in Switzerland. Front. Psychol. 5:1253. 10.3389/fpsyg.2014.01253 25408678 PMC4219388

[B28] Moore P. J. Mok M. M. C. Chan L. K. S. Lai P. Y. (2006). The Development of an indicator system for the affective and social schooling outcomes for primary and secondary students in Hong Kong. Educ. Psychol. 26, 273–301. 10.1080/01443410500344266

[B29] Noble T. McGrath H. (2015). PROSPER: a new framework for positive education. Psychol. Well Being 5:2 10.1186/s13612-015-0030-2

[B30] Norrish J. M. (2015a). The model for positive education, in Positive Education: The Geelong Grammar School Journey, ed Norrish J. M. (Oxford, UK: Oxford University Press), 29–50.

[B31] Norrish J. M. (2015b). Positive Education: The Geelong Grammar School Journey. Oxford: Oxford University Press.

[B32] Norrish J. M. Williams P. O'Connor M. Robinson J. (2013). An applied framework for positive education. Int. J. Wellbeing 3, 147–161. 10.5502/ijw.v3i2.2

[B33] Nunnally J. C. (1978). Psychometric Theory. New York, NY: McGraw-Hill.

[B34] OCED (2013). OECD Guidelines on Measuring Subjective well-being. OECD Publishing. 24600748

[B35] Oxford R. L. (2016). Powerfully positive: Searching for a model of language learner well-being, in Positive Psychology Perspectives on Foreign Language Learning and Teaching, eds Gabryś-Barker D. Gałajda D. (Basel: Springer International Publishing), 21–38.

[B36] Park N. Peterson C. (2006). Moral competence and character strengths among adolescents: the development and validation of the Values in Action Inventory of Strengths for Youth. J. Adolesc. 29, 891–909. 10.1016/j.adolescence.2006.04.011 16766025

[B37] Park N. Peterson C. Seligman M. E. P. (2006). Character strengths in fifty-four nations and the fifty US states. J. Posit. Psychol. 1, 118–129. 10.1080/17439760600619567

[B38] Peterson C. Seligman M. E. P. (2004). Character Strengths and Virtues: A handbook and Classification. Washington, DC: American Psychological Association.

[B39] Quinlan D. Swain N. Vella-Brodrick D. A. (2012). Character strengths interventions: building on what we know for improved outcomes. J. Happiness Stud. 13, 1145–1163. 10.1007/s10902-011-9311-5

[B40] Ryan R. M. Deci E. L. (2001). On happiness and human potentials: a review of research on hedonic and eudaimonic well-being. Annu. Rev. Psychol. 52, 141–166. 10.1146/annurev.psych.52.1.141 11148302

[B41] Ryff C. D. Keyes C. L. M. (1995). The structure of psychological well-being revisited. J. Pers. Soc. Psychol. 69, 719–727. 10.1037/0022-3514.69.4.719 7473027

[B42] Satorra A. Bentler P. M. (1994). Corrections to test statistics and standard errors in covariance structure analysis, in Latent Variables Analysis: Applications for Developmental Research (Thousand Oaks, CA, US: Sage Publications, Inc.), 399–419.

[B43] Schermelleh-Engel K. Moosbrugger H. Müller H. (2003). Evaluating the fit of structural equation models: Tests of significance and descriptive goodness-of-fit measures. Methods Psychol. Res. 8, 23–74.

[B44] Seligman M. E. P. (2011). Flourish: A Visionary New Understanding of Happiness and Well-Being (1st Free Press Hardcover ed.. ed.). New York, NY: Free Press.

[B45] Seligman M. E. Csikszentmihalyi M. (2000). Positive psychology: an introduction. Am. Psychol. 55, 5–14. 10.1037/0003-066X.55.1.5 11392865

[B46] Seligman M. E. P. Ernst R. M. Gillham J. Reivich K. Linkins M. (2009). Positive education: positive psychology and classroom interventions. Oxford Rev. Educ. 35, 293–311. 10.1080/03054980902934563

[B47] Shryack J. Steger M. F. Krueger R. F. Kallie C. S. (2010). The structure of virtue: an empirical investigation of the dimensionality of the virtues in action inventory of strengths. Pers. Individ. Dif. 48, 714–719. 10.1016/j.paid.2010.01.007

[B48] Toner E. Haslam N. Robinson J. Williams P. (2012). Character strengths and wellbeing in adolescence: structure and correlates of the Values in Action Inventory of Strengths for Children. Pers. Individ. Dif. 52, 637–642. 10.1016/j.paid.2011.12.014

[B49] Weber M. Wagner L. Ruch W. (2016). Positive feelings at school: on the relationships between students' character strengths, school-related affect, and school functioning. J. Happiness Stud. 17, 341–355. 10.1007/s10902-014-9597-1

[B50] Wheaton B. Muthén B. Alwin D. F. Summers G. F. (1977). Assessing reliability and stability in panel models. Sociol. Methodol. 8, 84–136. 10.2307/270754

[B51] White M. A. Murray A. S. Seligman M. E. P. (2015). Evidence-Based Approaches in Positive Education: Implementing a Strategic Framework for Well-Being in Schools. Dordrecht: Springer.

[B52] Wood A. M. Linley P. A. Maltby J. Kashdan T. B. Hurling R. (2011). Using personal and psychological strengths leads to increases in well-being over time: a longitudinal study and the development of the strengths use questionnaire. Pers. Individ. Dif. 50, 15–19. 10.1016/j.paid.2010.08.004

[B53] Wu G. K. Y. Mok M. M. C. (2017). Social and emotional learning and personal best goals in Hong Kong, in Social and Emotional Learning in Australia and the Asia-Pacific: Perspectives, Programs and Approaches (Singapore: Springer Nature), 219–231.

[B54] Yip P. S. F. (2016). Committee on Prevention of Student Suicides. Available online: http://www.edb.gov.hk/attachment/en/student-parents/crisis-management/about-crisis-management/CPSS_final_report_en.pdf

[B55] Zigmond A. S. Snaith R. P. (1983). The hospital anxiety and depression scale. Acta Psychiatr. Scand. 67, 361–370. 10.1111/j.1600-0447.1983.tb09716.x 6880820

