# Corrigendum: A Multidimensional PERMA-H Positive Education Model, General Satisfaction of School Life, and Character Strengths Use in Hong Kong Senior Primary School Students: Confirmatory Factor Analysis and Path Analysis Using the APASO-II

**DOI:** 10.3389/fpsyg.2018.01639

**Published:** 2018-09-19

**Authors:** Man K. Lai, Cynthia Leung, Sylvia Y. C. Kwok, Anna N. N. Hui, Herman H. M. Lo, Janet T. Y. Leung, Cherry H. L. Tam

**Affiliations:** ^1^Applied Social Sciences, Hong Kong Polytechnic University, Kowloon, Hong Kong; ^2^Department of Applied Social Sciences, City University of Hong Kong, Kowloon, Hong Kong

**Keywords:** APASO-II, Hong Kong, affective and social outcomes, character strengths, confirmatory factor analysis, path analysis, PERMA-H, positive education

In the original article, there was an error in the Funding statement. The correct Name for the Funder is Bei Shan Tang Foundation. The authors apologize for this error and state that this does not change the scientific conclusions of the article in any way.

The original article has been updated.

## Conflict of interest statement

The authors declare that the research was conducted in the absence of any commercial or financial relationships that could be construed as a potential conflict of interest.

